# Dietary diversity and its relationship with nutritional adequacy in 24 to 59 months old children in Iran: study protocol

**DOI:** 10.1186/s40795-022-00616-6

**Published:** 2022-10-23

**Authors:** Zahra Yari, Maryam Amini, Hamid Rasekhi, Bahareh Nikooyeh, Azam Doustmohammadian, Delaram Ghodsi, Samira Rabiei, Tirang R. Neyestani

**Affiliations:** 1 Department of Nutrition Research, National Nutrition and Food Technology Research Institute, Faculty of Nutrition Sciences and Food Technology, Shahid Beheshti University of Medical Science, No. 7., Hafezi St., Farahzadi Blvd, P.O. Box: 19395‐4741, Tehran, 1981619573 Iran; 2 Gastrointestinal and Liver Diseases Research Center (GILDRC), Iran University of Medical Sciences, Tehran, Iran

**Keywords:** Adequacy, Diet, Diversity, Nutrients, Children

## Abstract

**Background:**

Nutritional inadequacy, especially micronutrients, is still a serious concern among children in developing countries. Dietary diversity score (DDS) has been recommended as a proxy for nutritional adequacy. The present study aims to develop a questionnaire to assess the dietary diversity among 24–59 months children in Iran; also, the validity of the dietary diversity score as an indicator of nutritional status among the children will be assessed.

**Methods:**

In order to design the questionnaire, the Food and Agriculture Organization Guideline for measuring individual dietary diversity will be applied. According to the guideline, foods are categorized into nine groups, then dietary diversity score will be calculated by summing the number of food groups. In order to evaluate the efficacy of this questionnaire in predicting the nutritional status of the children, the relationship between dietary diversity score calculated by the questionnaire with nutritional adequacy, serum retinol and anthropometric indicators will be compared.

**Discussion:**

DDS can be a valuable indicator for predicting the adequacy of macronutrients and micronutrients specially in children. It is also significantly related to the mean probability of adequacy (MPA).

## Babkground

Adequate nutrition is essential for childhood growth and development and can protect children against both infectious and non-communicable diseases. To ensure the optimal nutritional status of the children, there is a need for continuous monitoring of their food intake [1]. Assessing food intake is time-consuming, costly, and labor-intensive [2]. Dietary diversity (DD) has been widely used as an indicator of nutritional adequacy, especially in developing countries. The simplicity of implementation and potential for large-scale use are two advantages of using dietary diversity compared to other food intake monitoring tools. The Food and Agriculture Organization (FAO) has recommended DD as the proxy for individuals’ nutritional adequacy, which can be implemented quickly, easily, and inexpensively [3].

Several studies have confirmed dietary diversity as a valuable indicator for predicting the adequacy of macronutrients or micronutrients in children [4–6]. A study conducted on adolescents reported that the nutrient adequacy ratio (NAR) in most of the studied micronutrients was positively correlated with the dietary diversity score (DDS) [7]. Another study found that DDS was significantly associated with the mean probability of adequacy (MPA) and the intake of most micronutrients [6]. Another study demonstrated that the DDS of 17 food groups was positively correlated with body mass index (BMI) and BMI for age Z-score of children equal to or younger than six years old. However, this correlation did not show similar results in those older than six years [8].

To the best of our knowledge, to date, no questionnaire has been developed to specifically assess the dietary diversity of children aged 24 to 59 months in Iran. Therefore, the present study aims to develop a questionnaire to assess the dietary diversity of children aged 24 to 59 months in Iran. Furthermore, the relationship between DDS with nutritional adequacy indices, serum retinol levels, and anthropometric measurements among children aged 24—59 months in eleven provinces of Iran will be calculated.

## Methods and design

### Developing the dietary diversity questionnaire

Guided by FAO Guideline for measuring individual dietary diversity [3, 9], scale items will be generated. According to the guideline, as Table 1 shows, foods are categorized into nine groups. Food quantities of at least one tablespoon (≥ 15 g) will be included in the score. Dietary diversity scores will be calculated by summing the number of food groups consumed by the individual respondents.

**Table 1:** Food groupings for calculation of dietary diversity score

**No**	C**omponents/name of the food group**
1	**Cereals and starchy vegetables** Corn kernels, wheat, rice, flour, and any food that is produced with them (such as bread, pasta), potatoes, peas, and beans
2	**Dark green leafy vegetables** Different types of raw and cooked dark green leafy vegetables such as spinach, parsley, leeks, and watercress
3	**Vegetables and fruits rich in vitamin A** Yellow and orange vegetables and fruits and their completely natural juices such as carrots, cantaloupe, squash, mango, and apricots
4	**Other vegetables and fruits** Including tomatoes, onions, cucumbers, and eggplants
5	**Meat and fish** Different types of red meat, fish, and poultry
6	**Organ meats** Including heart, liver, and kidney
7	**Eggs**
8	**Milk and dairy products** Different types of milk, cheese, yogurt, and other milk products
9	**Legumes, seeds, and nuts** Legumes, nuts, seeds, and foods made with them

### Content validity

Experts in nutrition will evaluate the first version of the questionnaire regarding its relevance, clarity, meaningfulness and completeness.

### Predictive validity

To analyze any correlation between DDS and NARs, mean adequacy ration (MAR), anthropometric indicators (Z scores of height for age, weight for age, and BMI for age), and serum retinol, Pearson correlation analysis will be conducted.

### Sample size

To calculate the sample size, α and β errors were considered to be 5% and 10%, respectively. According to Hatløy [10], the correlation coefficient between MAR and DDS was estimated to be 0.4. The sample size was calculated as 51 children in each province.

### Recruitment and eligibility screening

Children aged 24–59 months from eleven provinces, including Sistan-Baloochestan, South Khorasan, Ilam, Kohkiluyeh-Boyerahmad, Khuzestan, Kerman, Hormozgan, Bushehr, Kordestan, North Khorasan, and Tehran will be included in this study. Exclusion criteria include reluctance to participate in the study, physical and/or mental retardation, and adherence to special diets. Eligible children will be enrolled through random cluster sampling based on proportion to size. The Statistics Center of Iran will provide the list of clusters. Ten clusters, including seven urban and three rural clusters, will be randomly selected in each province. A total of 561 children will be enrolled.

### Data collection

We will use a mobile application and an electronic questionnaire to collect information. General information will be obtained using a questionnaire. The weight and height of the children will be measured by standard methods. Biochemical testing will be performed for each child Serum retinol assessments will be performed in the same laboratory using high-performance liquid chromatography (HPLC). All parents/caregivers should sign an informed consent form after a full review of the inclusion and exclusion criteria and an explanation of the study protocol. The study protocol has been approved by the Ethical Committees of National Nutrition and Food Technology Research Institute, Shahid Beheshti University of Medical Sciences.

### Nutritional assessment

The dietary diversity questionnaire will be completed by the children's parents/caregivers. Also, in order to evaluate the intake of energy and nutrients, two 24-h food recalls will be completed. The interval between the two recalls is one to a maximum of 7 days. The consumed food will first be converted to grams, then the nutrients and energy contents will be calculated using the Nutritionist-IV software and United States Department of Agriculture (USDA) food composition table (FCT). An Iranian FCT will also be used for local food items. All phases will be performed by experienced and trained nutritionists.

### Nutrient adequacy

To estimate the nutrient adequacy, Nutrient Adequacy Ratio (NAR) for the energy, protein, and micronutrients (vitamin A, vitamin E, B1, B2, niacin, B6, B12, folate, vitamin C, calcium, iron, and zinc) will be calculated [10]. As an overall measure of nutrient adequacy, MAR will be calculated, which is the sum of each NAR (truncated at 100%) divided by the number of nutrients (excluding energy and protein) [10]. For both NAR and MAR, a value of 100% is ideal since it means that the intake is the same as the requirement. The energy, protein, vitamins, and minerals requirement for each sex and age group will be calculated based on the Estimated Average Requirements (EAR) [11–13].

### Data analysis

Data will be analyzed using STATA software (version 17; StataCorp). A *P* value < 0.05 will be considered statistically significant for all analyses. To analyze anthropometric data, the WHO Anthro Survey Analyzer will be applied. The data file in a comma-delimited format (.csv) will be uploaded to the URL: https://worldhealthorg.shinyapps.io/anthro/. After calculating DDS, to analyze any correlation between DDS and NARs, MAR, anthropometric indicators (Z scores of height for age, weight for age, and BMI for age), and serum retinol, Pearson correlation analysis will be conducted.

## Discussion

To our knowledge, no questionnaire has been specifically designed to assess the dietary diversity of children aged 24 to 59 months in Iran. The dietary diversity score, compared with other food consumption indicators, has been defined as a practical tool, especially in developing countries [1]. It has been reported that DDS can be a valuable indicator for predicting the adequacy of macronutrients and micronutrients in children [4–6]. A study in adolescents showed that the NAR of many micronutrients increased with the DDS, indicating a positive correlation between nutrient adequacy and the DDS [5]. It has also been found that DDS is significantly related to the MPA and also to the intake of most micronutrients [6].

Despite various dietary guidelines recommending the consumption of a variety of foods to meet nutritional needs, the question remains on how to develop an indicator to measure nutritional diversity and adequacy. Given that the current approach to assessing nutritional adequacy depends on difficult and time-consuming measurements, there is a need to develop a convenient and cost-effective tool. Since the relationship between diversity and nutritional adequacy has been observed in other age and demographic groups, it seems that this questionnaire can reasonably predict nutritional adequacy in Iranian children aged 24–59 months. If our results confirm this hypothesis, it will be a novel, useful option for predicting the dietary diversity and nutritional status of children to ensure their nutritional adequacy.

## Data Availability

Since it is a protocol study no data will be produced.

## Abbreviations

DDS: Dietary diversity score
MPA: Mean probability of adequacy
FAO: Food and Agriculture Organization
DD: Dietary diversity
NAR: Nutrient adequacy ratio
BMI: Body mass index
MAR: Mean adequacy ratio
HPLC: High-performance liquid chromatography
USDA: United States Department of Agriculture
FCT: Food composition table
EAR: Estimated Average Requirements
WHO: World Health Organization
